# A Roadmap for Boosting Model Generalizability for Predicting Hospital Encounters for Asthma

**DOI:** 10.2196/33044

**Published:** 2022-03-01

**Authors:** Gang Luo

**Affiliations:** 1 Department of Biomedical Informatics and Medical Education University of Washington Seattle, WA United States

**Keywords:** clinical decision support, forecasting, machine learning, patient care management, medical informatics, asthma, health care, health care systems, health care costs, prediction models, risk prediction

## Abstract

In the United States, ~9% of people have asthma. Each year, asthma incurs high health care cost and many hospital encounters covering 1.8 million emergency room visits and 439,000 hospitalizations. A small percentage of patients with asthma use most health care resources. To improve outcomes and cut resource use, many health care systems use predictive models to prospectively find high-risk patients and enroll them in care management for preventive care. For maximal benefit from costly care management with limited service capacity, only patients at the highest risk should be enrolled. However, prior models built by others miss >50% of true highest-risk patients and mislabel many low-risk patients as high risk, leading to suboptimal care and wasted resources. To address this issue, 3 site-specific models were recently built to predict hospital encounters for asthma, gaining up to >11% better performance. However, these models do not generalize well across sites and patient subgroups, creating 2 gaps before translating these models into clinical use. This paper points out these 2 gaps and outlines 2 corresponding solutions: (1) a new machine learning technique to create cross-site generalizable predictive models to accurately find high-risk patients and (2) a new machine learning technique to automatically raise model performance for poorly performing subgroups while maintaining model performance on other subgroups. This gives a roadmap for future research.

## Introduction

### Asthma Care Management and Our Prior Work on Predictive Modeling

In the United States, ~9% of people have asthma [1-3]. Each year, asthma incurs US$ 56 billion of health care cost [4] and many hospital encounters covering 1.8 million emergency room visits and 439,000 hospitalizations [1]. As is the case with many chronic diseases, a small percentage of patients with asthma use most health care resources [5,6]. The top 1% of patients spend 25% of the health care costs. The top 20% spend 80% [5,7]. An effective approach is urgently in need to prospectively identify high-risk patients and intervene early to avoid health decline, improve outcomes, and cut resource use. Most major employers purchase and nearly all private health plans offer care management services for preventive care [8-10]. Care management is a collaborative process to assess, coordinate, plan, implement, evaluate, and monitor the services and options to meet the health and service needs of a patient [11]. A care management program employs care managers to call patients regularly to assess their status, arrange doctor appointments, and coordinate health-related services. Proper use of care management can cut down hospital encounters by up to 40% [10,12-17]; lower health care cost by up to 15% [13-18]; and improve patient satisfaction, quality of life, and adherence to treatment by 30%-60% [12]. Care management can cost >US$ 5000 per patient per year [13] and normally enrolls no more than 3% of patients [7] owing to resource limits.

Correctly finding high-risk patients to enroll is crucial for effective care management. Currently, the best method to identify high-risk patients is to use models to predict each patient’s risk [19]. Many health plans such as those in 9 of 12 metropolitan communities [20] and many health care systems [21] use this method for care management. For patients predicted to have the highest risk, care managers manually review patients’ medical records, consider factors such as social dimensions, and make enrollment decisions. However, prior models built by others miss >50% of true highest-risk patients and mislabel many low-risk patients as high risk [5,12,22-36]. This makes enrollment align poorly with patients who would benefit most from care management [12], leading to suboptimal care and higher costs. As the patient population is large, a small boost in model performance will benefit many patients and produce a large positive impact. Of the top 1% patients with asthma who would incur the highest costs, for every 1% more whom one could find and enroll, one could save up to US$ 21 million more in asthma care every year as well as improve outcomes [5,26,27].

To address the issue of low model performance, we recently built 3 site-specific models to predict whether a patient with asthma would incur any hospital encounter for asthma in the subsequent 12 months, 1 model for each of the 3 health care systems—the University of Washington Medicine (UWM), Intermountain Healthcare (IH), and Kaiser Permanente Southern California (KPSC) [21,37,38]. Each prior model that others built for a comparable outcome [5,26-34] had an area under the receiver operating characteristic curve (AUC) that was ≤0.79 and a sensitivity that was ≤49%. Our models raised the AUC to 0.9 and the sensitivity to 70% on UWM data [21], the AUC to 0.86 and the sensitivity to 54% on IH data [37], and the AUC to 0.82 and the sensitivity to 52% on KPSC data [38].

Our eventual goal is to translate our models into clinical use. However, despite major progress, our models do not generalize well across sites and patient subgroups, and 2 gaps remain.

### Gap 1: The Site-Specific Models Have Suboptimal Generalizability When Applied to the Other Sites

Each of our models was built for 1 site. As is typical in predictive modelling [39,40], when applied to the other sites, the site-specific model had AUC drops of up to 4.1% [38], potentially degrading care management enrollment decisions. One can do transfer learning using other source health care systems' raw data to boost model performance for the target health care system [41-45], but health care systems are seldom willing to share raw data. Research networks [46-48] mitigate the problem but do not solve it. Many health care systems are not in any network. Health care systems in the network share raw data of finite attributes. Our prior model-based transfer learning approach [49] requires no raw data from other health care systems. However, it does not control the number of features (independent variables) used in the final model for the target site, creating difficulty to build the final model for the target site for clinical use. Consequently, it is never implemented in computer code.

### Gap 2: The Models Exhibit Large Performance Gaps When Applied to Specific Patient Subgroups

Our models performed up to 8% worse on Black patients. This is a typical barrier in machine learning, where many models exhibit large subgroup performance gaps, for example, of up to 38% [50-57]. No existing tool for auditing model bias and fairness [58,59] has been applied to our models. Currently, it is unknown how our models perform on key patient subgroups defined by independent variables such as race, ethnicity, and insurance type. In other words, it is unknown how our models perform for different races, different ethnicities, and patients using different types of insurance. Large performance gaps among patient subgroups can lead to care inequity and should be avoided.

Many methods to improve fairness in machine learning exist [50-52]. These methods usually boost model performance on some subgroups at the price of lowering both model performance on others and the overall model performance [50-52]. Lowering the overall model performance is undesired [51,57]. Owing to the large patient population, even a 1% drop in the overall model performance could potentially degrade many patients’ outcomes. Chen et al [57] cut model performance gaps among subgroups by collecting more training data and adding additional features, both of which are often difficult or infeasible to do. For classifying images via machine learning, Goel et al’s method [55] raised the overall model performance and cut model performance gaps among subgroups of a value of the dependent variable—not among subgroups defined by independent variables. The dependent variable is also known as the outcome or the prediction target. An example of the dependent variable is whether a patient with asthma will incur any hospital encounter for asthma in the subsequent 12 months. The independent variables are also known as features. Race, ethnicity, and insurance type are 3 examples of independent variables. Many machine learning techniques to handle imbalanced classes exist [60,61]. In these techniques, subgroups are defined by the dependent variable rather than by independent variables.

### Contributions of This Paper

To fill the 2 gaps on suboptimal model generalizability and let more high-risk patients obtain appropriate and equitable preventive care, the paper makes 2 contributions, thereby giving a roadmap for future research.

1. To address the first gap, a new machine learning technique is outlined to create cross-site generalizable predictive models to accurately find high-risk patients. This is to cut model performance drop across sites.
2. To address the second gap, a new machine learning technique is outlined to automatically raise model performance for poorly performing subgroups while maintaining model performance on other subgroups. This is to cut model performance gaps among patient subgroups and to reduce care inequity.

The following sections describe the main ideas of the proposed new machine learning techniques.

## Machine Learning Technique for Creating Cross-Site Generalizable Predictive Models to Accurately Find High-risk Patients

### Our Prior Models

In our prior work [21,37,38], for each of the 3 health care systems (sites), namely, KPSC, IH, and UWM, >200 candidate features were checked and the site’s data were used to build a full site-specific extreme gradient boosting (XGBoost) model to predict hospital encounters for asthma. XGBoost [62] automatically chose the features to be used in the model from the candidate features, computed their importance values, and ranked them in the descending order of these values. The top (~20) features with importance values ≥1% have nearly all of the predictive power of all (on average ~140) features used in the model [21,37,38]. Although some lower-ranked features are unavailable at other sites, each top feature such as the number of patient’s asthma-related emergency room visits in the prior 12 months is computed using (eg, diagnosis, encounter) attributes routinely collected by almost every American health care system that uses electronic medical records. Using the top features and the site’s data, a simplified XGBoost model was built. It, but not the full model, can be applied to other sites. The simplified model performed similarly to the full model at the site. However, when applied to another site, even after being retrained on its data, the simplified model performed up to 4.1% worse than the full model built specifically for it, as distinct sites have only partially overlapping top features [21,37,38].

### Building Cross-Site Generalizable Models

To ensure that the same variable is called the same name at different sites and the variable’s content is recorded in the same way across these sites, the data sets at all source sites and the target site are converted into the Observational Medical Outcomes Partnership (OMOP) common data model [63] and its linked standardized terminologies [64]. If needed, the data model is extended to cover the variables that are not included in the original data model but exist in the data sets. 

**Figure 1:**
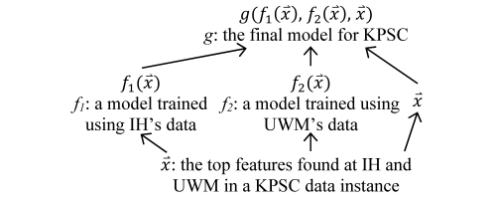
The method used in this study to build cross-site generalizable models. IH: Intermountain Healthcare. KPSC: Kaiser Permanente Southern California. UWM: University of Washington Medicine.

Our goal is to build cross-site generalizable models fulfilling 2 conditions. First, the model uses a moderate number of features. Controlling the number of features used in the model would ease the future clinical deployment of the model. Second, a separate component or copy of the model is initially built at each source site. When applied to the target site and possibly after being retrained on its data, the model performs similarly to the full model built specifically for it. To reach our goal for the case of IH and UWM being the source sites and KPSC being the target site, we proceed in 2 steps (Figure 1). In step 1, the top features found at each source site are combined. For each source site, the combined top features, its data, and the machine learning algorithm adopted to build its full model are used to build an expanded simplified model. Compared with the original simplified model built for the site, the expanded simplified model uses more features with predictive power and tends to generalize better across sites. In step 2, model-based transfer learning is conducted to further boost model performance. For each data instance of the target site, each source site’s expanded simplified model is applied to the data instance, a prediction result is computed, and the prediction result is used as a new feature. For the target site, its data, the combined top features found at the source sites, and the new features are used to build its final model.

To reach our goal for the case that IH or UWM is the target site and KPSC is one of the source sites, we need to address the issue that the claim-based features used at KPSC [38] are unavailable at IH, UWM, and many other health care systems with no claim data. At KPSC, these features are dropped and the other candidate features are used to build a site-specific model and recompute the top features. This helps reach the effect that the top features found at each of KPSC, IH, and UWM are available at all 3 sites and almost every other American health care system that uses electronic medical record systems. In the unlikely case that any recomputed top feature at KPSC violates this, the feature is skipped when building cross-site generalizable models.

Our method to build cross-site generalizable models can handle all kinds of prediction targets, features, and models used at the source and target sites. Given a distinct prediction target, if some top features found at a source site are unavailable at many American health care systems using electronic medical record systems, the drop→recompute→skip approach shown above can be used to handle these features. Moreover, at any source site, if the machine learning algorithm used to build the full site-specific model is like XGBoost [62] or random forest that automatically computes feature importance values, the top features with the highest importance values can be used. Otherwise, if the algorithm used to build the full model does not automatically compute feature importance values, an automatic feature selection method [65] like the information gain method can be used to choose the top features. Alternatively, XGBoost or random forest can be used to build a model, automatically compute feature importance values, and choose the top features with the highest importance values.

Our new model-based transfer learning approach waives the need for source sites’ raw data. Health care systems are more willing to share with others trained models than raw data. A model trained using the data of a source site contains much information that is useful for the prediction task at the target site. This information offers much value when the target site has insufficient data for model training. If the target site is large, this information can still be valuable. Distinct sites have differing data pattern distributions. A pattern that matches a small percentage of patients and is difficult to identify at the target site could match a larger percentage of patients and be easier to identify at one of the source sites. In this case, its expanded simplified model could incorporate the pattern through model training to better predict the outcomes of certain types of patients, which is difficult to do using only the information from the target site but no information from the source sites. Thus, we expect that compared with just retraining a source site’s expanded simplified model on the target site’s data, doing model-based transfer learning in step 2 could lead to a better performing final model for the target site. 

When the target site goes beyond IH, UWM, and KPSC, IH, UWM, and KPSC can be used as the source sites to have more top features to combine. This would make our cross-site models generalize even better.

## Machine Learning Technique for Automatically Raising Model Performance for Poorly Performing Patient Subgroups While Maintaining Model Performance on Other Subgroups to Reduce Care Inequity

Several clinical experts are asked to identify several patient subgroups of great interest to clinicians (eg, by race, ethnicity, insurance type) through discussion. These subgroups are not necessarily mutually exclusive of each other. Each subgroup is defined by one or more attribute values. Given a predictive model built on a training set, model performance on each subgroup on the test set is computed and shown [58,59]. Machine learning needs enough training data to work well. Often, the model performs much worse on a small subgroup than on a large subgroup [50,52]. After identifying 1 or more target subgroups where the model performs much worse than on other subgroups [51], a new dual-model approach is used to raise model performance on the target subgroups while maintaining model performance on other subgroups.

**Figure 2:**
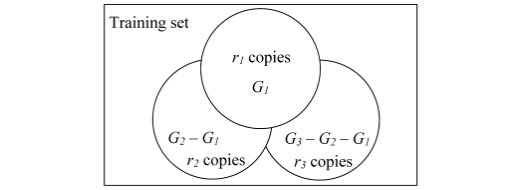
Oversampling for 3 target patient subgroups *G_1_*, *G_2_*, and *G_3_*.

More specifically, given *n* target patient subgroups, they are sorted as *G_i_* (1≤*i*≤*n*) in ascending order of size and oversampled based on *n* integers *r_i_* (1≤*i*≤*n*) satisfying *r_1_*≥*r_2_*≥…≥*r_n_*>1. As Figure 2 shows, for each training instance in *G_1_*, *r_1_* copies of it including itself are made. For each training instance in 
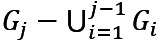
 (2≤*j*≤*n*), *r_j_* copies of it, including itself, are made. Intuitively, the smaller the *i* (1≤*i*≤*n*) and thus *G_i_*, the more aggressive oversampling is needed on *G_i_* for machine learning to work well on it. The sorting ensures that if a training instance appears in ≥2 target subgroups, copies are made for it based on the largest *r_i_* of these subgroups. If needed, 1 set of *r_i_*’s could be used for training instances with bad outcomes, and another set of *r_i_*’s could be used for training instances with good outcomes [66]. 
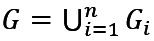
 is the union of the *n* target subgroups. Using the training instances outside *G*, the copies made for the training instances in *G* and an automatic machine learning model selection method like our formerly developed one [67], the AUC on *G* is optimized, the values of *r_i_* (1≤*i*≤*n*) are automatically selected, and a second model is trained. As is typical in using oversampling to improve fairness in machine learning, compared with the original model, the second model tends to perform better on *G* and worse on the patients outside *G* [51,66] because oversampling increases the percentage of training instances in *G* and decreases the percentage of training instances outside *G*. To avoid running into the case of having insufficient data for model training, no undersampling is performed on the training instances outside *G*. The original model is used to make predictions on the patients outside *G*. The second model is used to make predictions on the patients in *G*. In this way, model performance on *G* can be raised without lowering either model performance on the patients outside *G* or the overall model performance. All patients’ data instead of only the training instances in *G* are used to train the second model. Otherwise, the second model may perform poorly on *G* owing to insufficient training data in *G* [51]. For a similar reason, we choose to not use decoupled classifiers, where a separate classifier is trained for each subgroup by using only that subgroup’s data [51] on the target subgroups [57].

**Figure 3:**
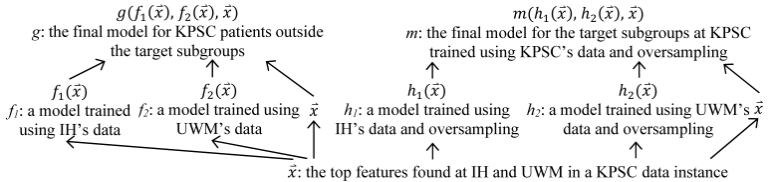
The method used in this study to boost a cross-site generalizable model’s performance on the target patient subgroups. IH: Intermountain Healthcare. KPSC: Kaiser Permanente Southern California. UWM: University of Washington Medicine.

The above discussion focuses on the case that the original model is built on 1 site’s data without using any other site’s information. When the original model is a cross-site generalizable model built for the target site using the method in the “Building cross-site generalizable models” section and models trained at the source sites, to raise model performance on the target patient subgroups, we change the way to build the second model for the target site by proceeding in 2 steps (Figure 3). In step 1, the top features found at each source site are combined. Recall that *G* is the union of the *n* target subgroups. For each source site, the target subgroups are oversampled in the way mentioned above; the AUC on *G* at the source site is optimized; and its data both in and outside *G*, the combined top features, and the machine learning algorithm adopted to build its full model are used to build a second expanded simplified model. In step 2, model-based transfer learning is conducted to incorporate useful information from the source sites. For each data instance of the target site, each source site’s second expanded simplified model is applied to the data instance, a prediction result is computed, and the prediction result is used as a new feature. For the target site, the target subgroups are oversampled in the way mentioned above, the AUC on *G* at the target site is optimized, and its data both in and outside *G*, the combined top features found at the source sites, and the new features are used to build the second model for it. For each *i* (1≤*i*≤*n*), each of the source and target sites could use a distinct oversampling ratio *r_i_*.

## Discussion

Predictive models differ by diseases and other factors. However, our proposed machine learning techniques are general and depend on no specific disease, patient cohort, or health care system. Given a new data set with a differing prediction target, disease, patient cohort, set of health care systems, or set of variables, one can use our proposed machine learning techniques to improve model generalizability across sites, as well as to boost model performance on poorly performing patient subgroups while maintaining model performance on others. For instance, our proposed machine learning techniques can be used to improve model performance for predicting other outcomes such as adherence to treatment [68] and no-shows [69]. This will help target resources such as interventions to improve adherence to treatment [68] and reminders by phone calls to reduce no-shows [69]. Care management is widely adopted to manage patients with chronic obstructive pulmonary disease, patients with diabetes, and patients with heart disease [6], where our proposed machine learning techniques can also be used. Our proposed predictive models are based on the OMOP common data model [63] and its linked standardized terminologies [64], which standardize administrative and clinical variables from at least 10 large health care systems in the United States [47,70]. Our proposed predictive models apply to those health care systems and others using OMOP.

## Conclusions

To better identify patients likely to benefit most from asthma care management, we recently built the most accurate models to date to predict hospital encounters for asthma. However, these models do not generalize well across sites and patient subgroups, creating 2 gaps before translating these models into clinical use. This paper points out these 2 gaps and outlines 2 corresponding solutions, giving a roadmap for future research. The principles of our proposed machine learning techniques generalize to many other clinical predictive modeling tasks.

## Abbreviations

AUC: area under the receiver operating characteristic curve
IH: Intermountain Healthcare
KPSC: Kaiser Permanente Southern California
OMOP: Observational Medical Outcomes Partnership
UWM: University of Washington Medicine
XGBoost: extreme gradient boosting
